# Multi-Target Molecular Detection of Sexually Transmitted Infections in Women Living with HIV in Northeastern Brazil

**DOI:** 10.3390/tropicalmed10120354

**Published:** 2025-12-18

**Authors:** Melina Vieira Alves, Letícia Alves dos Santos Silva, Maria Luísa Rodrigues Nolasco, Anny Beatriz de Oliveira Gama, Márcia Guimarães da Silva, Marcus Vinicius de Aragão Batista

**Affiliations:** 1 Laboratory of Molecular Genetics and Biotechnology (GMBio), Department of Biology, Center for Biological and Health Sciences, Federal University of Sergipe, Sao Cristovao 49107-230, SE, Brazil; melinavieiraalves12@gmail.com (M.V.A.); leti_alvess@hotmail.com (L.A.d.S.S.); luisanolasco1@gmail.com (M.L.R.N.); annygama019@gmail.com (A.B.d.O.G.); 2 Department of Pathology, Botucatu Medical School, São Paulo State University, Botucatu 18618-687, SP, Brazil; marcia.guimaraes@unesp.br

**Keywords:** molecular diagnosis, HPV, HIV, *Chlamydia trachomatis*, *Trichomonas vaginalis*, *Neisseria gonorrhoeae*

## Abstract

Co-infection by human papillomavirus (HPV) and human immunodeficiency virus (HIV) facilitates cervical carcinogenesis, and additional cofactors such as other sexually transmitted infections (STI) further aggravate this scenario. This study aimed to validate a molecular detection strategy for *Chlamydia trachomatis*, *Trichomonas vaginalis* and *Neisseria gonorrhoeae* and to assess the association of these infections with cervical lesions in HPV-positive women living with HIV in Northeastern Brazil. In total, 155 samples were collected from CRIST/AIDS. After microorganism detection by conventional PCR, a multiplex PCR was standardized and validated. A prevalence of 9.03% was observed for *C. trachomatis* and 6.45% for *T. vaginalis*, with 0.64% co-infection. In addition, infection with both STIs showed a prevalence of 0.64%. Among HPV-positive women, high-risk genotypes accounted for 70.9% of cases, with HPV-16 being the most prevalent (35.5%). Overall, 18.2% of women presented cervical lesions, and 13.2% of those with co-detection of *C. trachomatis* and *T. vaginalis* were associated with high-grade squamous intraepithelial lesions (HSIL). These findings highlight the clinical relevance of screening for multiple STIs in HPV-positive women living with HIV and support the incorporation of multiplex molecular testing into public health strategies to improve early detection and targeted management.

## 1. Introduction

Cervical cancer ranks as the fourth most common malignancy among women globally [1], with human papillomavirus (HPV) recognized as its primary etiological agent [2]. HPV is one of the most prevalent sexually transmitted infections (STIs) worldwide [3] and is directly implicated in the development of precancerous lesions and malignant neoplasms across various anatomical sites [2]. The presence of human immunodeficiency virus (HIV) further exacerbates this scenario [4], as women living with HIV are at significantly increased risk for persistent HPV infection and progression to high-grade lesions due to immunosuppression [5]. Notably, HPV infection rates in this population can be up to six times higher than in HIV-negative women [5,6], and treatment outcomes tend to be poorer, regardless of the therapeutic modality employed [7].

In 2018, approximately 6% of all new cervical cancer cases worldwide were diagnosed in women living with HIV. This proportion varied considerably across regions, ranging from less than 5% to over 40% [8]. A meta-analysis by Da Silva et al. [9] reported an overall HPV prevalence of 62% among women with HIV in Brazil, of which 40% corresponded to high-risk HPV types. The study demonstrated that HIV-positive women exhibit substantially higher rates of high-risk HPV infection compared with HIV-negative women. Specifically, the prevalence of high-risk HPV was 43% versus 21% in the Southeast, 24% versus 11% in the South, 36% versus 18% in the North, and 37% versus 23% in the Northeast. These findings underscore the disproportionate burden of oncogenic HPV types among women living with HIV across all Brazilian regions [9].

In addition to the higher prevalence observed in Brazil, a systematic review conducted by Tadese et al. [5] indicates that this increased susceptibility among people living with HIV also translates into a greater risk of developing HPV-related precancerous lesions and invasive cancers. Among women with HIV, premalignant cervical lesions are more prevalent, more likely to persist, and have a higher recurrence rate compared to the general population [10]. High HIV viral load and persistent infection with oncogenic HPV types are key factors associated with progression to precancerous lesions [11].

Other sexually transmitted microorganisms, such as *Neisseria gonorrhoeae*, *Chlamydia trachomatis*, and *Trichomonas vaginalis*, also play a significant role in this context, acting as cofactors that exacerbate local inflammation, increase susceptibility to HIV infection [12,13], and contribute to cervical carcinogenesis. These pathogens are among the most prevalent curable STIs worldwide and frequently remain asymptomatic in women, which facilitates underdiagnosis and ongoing transmission [14]. In Brazil, several studies have investigated HIV and STIs co-infection. Reported STIs prevalence varies according to factors such as the specific infectious agent involved, the characteristics of the population studied, and the diagnostic methods employed [13,15,16,17,18]. Given their heightened vulnerability to persistent infections and neoplastic progression, women living with HIV require more rigorous and frequent screening protocols for cervical cancer prevention and early detection [7]. However, in low- and middle-income countries, limited access to laboratory tests has led the World Health Organization (WHO) to recommend syndromic management of STIs, which relies on the clinical identification of signs and symptoms rather than laboratory confirmation [19,20].

Conventional diagnostic methods, such as cell culture, microscopic examination, enzyme immunoassay, and other serological techniques, still have limitations [21,22,23]. These methods are generally time-consuming, labor-intensive, and often lack the sensitivity required for the reliable detection of STIs, particularly in asymptomatic individuals [24,25]. In contrast, molecular diagnostic techniques have gained prominence for the detection of STI-related pathogens due to their higher sensitivity and specificity [26]. Methods such as conventional PCR, real-time PCR, multiplex PCR, and microarray assays provide quicker results while enhancing diagnostic precision compared to traditional approaches [23,27,28,29,30,31].

Therefore, several commercial kits and systems based on nucleic acid amplification tests (NAATs) have been developed for the detection of STIs [32,33,34,35]. However, these systems still have limitations, including high cost, low sample throughput, and the restricted number of pathogens detectable in a single assay [28]. These constraints highlight the need for the development of sensitive, multiplex, and high-throughput diagnostic methods capable of simultaneously detecting multiple STI pathogens [36]. Ideally, such methods should also offer rapid and accurate results at a low cost, thereby enhancing accessibility, enabling timely treatment, and ultimately generating a broader impact on public health outcomes [37,38]. Therefore, the adoption of more accurate diagnostic strategies is crucial, especially among women living with HIV, who face a higher risk of co-infections and adverse clinical outcomes.

In this context, the multiplex molecular method aims to meet this need by simultaneously detecting *N. gonorrhoeae*, *C. trachomatis*, and *T. vaginalis* with high sensitivity, specificity, and accuracy, even at low pathogen loads. Furthermore, its ability to identify coinfections enables a more comprehensive assessment of their relationship with cervical lesions in HPV-positive women, thereby filling an important diagnostic gap and contributing to earlier intervention and improved clinical management in this vulnerable population. Thus, the aim of this study was to validate a molecular method for simultaneously detect *Neisseria gonorrhoeae*, *Chlamydia trachomatis*, and *Trichomonas vaginalis*, and to investigate the association of these infections with the development of cervical lesions in HPV-positive women living with HIV in Northeastern Brazil.

## 2. Materials and Methods

### 2.1. Study Population and Ethical Considerations

The study population consisted of 155 women living with HIV admitted to the reference center for sexually transmitted infections, HIV and AIDS (CRIST/AIDS) located in the state of Sergipe, Northeastern Brazil, between 2014 and 2017, who tested positive for HPV. Some epidemiological aspects of human papillomavirus infection in this population have been analyzed in a previous study [39]. Ethical approval was obtained from the Research Ethics Committee of the Federal University of Sergipe (protocol number: CAAE 92514618.8.0000.5546). Informed consent was obtained from all of the participants, who subsequently signed an informed consent form. Each participant completed a questionnaire with sociodemographic questions, and cervical content was collected using a sterile plastic gynecological brush. Cervical samples were stored in PBS solution (pH 7.4) and kept at −20 °C until analysis.

### 2.2. Eligibility Criteria

The recruited participants were women living with HIV who attended the referral clinic during the study period. The study included women who provided adequate cervical samples for molecular testing and who tested positive for HPV, in accordance with the research focus. Participants whose biological samples were insufficient or degraded for DNA extraction or amplification, those with missing information for key sociodemographic, clinical, or behavioral variables, or those who had undergone a previous total hysterectomy were not included in the corresponding analyses. Women who refused to participate or withdrew their consent at any stage were also not included.

### 2.3. Molecular Detection of HPV

For DNA extraction from cervical samples, the Wizard^®^ Genomic DNA Purification Kit (Promega Corporation, Madison, WI, USA) was used, according to the manufacturer’s instructions. The samples were tested for HPV using PCR and nested-PCR with the primers MY11/09 [40] and GP5+/6+ [41], respectively.

For the first reaction, 3 µL of extracted DNA, 10 µL of PCR Master Mix, 0.5 µL of MgCl_2_, 5.5 µL of H_2_O, and 0.5 µL of each primer were used. Ultrapure water was used as a negative control and clinical samples positive for HPV were used as positive controls. The PCR cycles were: initial denaturation at 95 °C for 5 min, 40 cycles of 95 °C for 45 s, annealing at 50 °C for 45 s, extension at 72 °C for 45 s, and a final extension at 72 °C for 5 min.

For nested PCR, the same volumes of reagents and the amplification product obtained in the previous reaction were used. The PCR cycles were: initial denaturation at 95 °C for 5 min, 40 cycles of 95 °C for 45 s, annealing at 47.7 °C for 30 s, extension at 72 °C for 30 s, and a final extension at 72 °C for 5 min. The amplified products were visualized by electrophoresis in 1.5% agarose gel.

### 2.4. Molecular Detection of C. trachomatis, T. vaginalis and N. gonorrhoeae

The standardization of conventional PCR for the detection of *C. trachomatis* was based on the protocol proposed by Marconi et al. [42], with modifications. 4 µL of extracted DNA, 10 µL of PCR Master Mix, 1 µL of MgCl_2_, 4.2 µL of H_2_O, and 0.2 µL of each primer were added (Table 1). Ultrapure water was used as a negative control, and clinical samples positive for *C. trachomatis* were used as positive controls. The PCR cycles were: initial denaturation at 95 °C for 5 min, 40 cycles of 95 °C for 30 s, annealing at 55 °C for 30 s, extension at 72 °C for 30 s, and a final extension at 72 °C for 5 min. Subsequently, PCR products were subjected to electrophoresis in 2% agarose gel. Positive samples were purified using the Wizard^®^ SV Gel and PCR Clean-UP System Kit (Promega Corporation, Madison, WI, USA), according to the manufacturer’s instructions. The samples were sequenced using the BigDye Terminator v3.1 Cycle Sequencing Kit (Thermo Scientific, Waltham, MA, USA).

The protocol proposed by Gimenes et al. [44] was used to perform the detection of *T. vaginalis* and *N. gonorrhoeae*. For each sample, 0.5 μL of MgCl_2_, 2 μL of H_2_O, 10 μL of PCR Master Mix, 0.5 μL of each primer, and 1.5 μL of DNA were added. Ultrapure water was used as a negative control, and clinical samples positive for *T. vaginalis* and *N. gonorrhoeae* were used as positive controls. The parameters used in PCR were: initial denaturation at 94 °C for 5 min; 35 cycles at 94 °C for 45 s, annealing at 62 °C for 30 s, extension at 72 °C for 30 s, and ending at 72 °C for 5 min for final extension. The PCR product was then subjected to electrophoresis in 1.5% agarose gel. Positive samples were purified using the Wizard^®^ SV Gel and PCR Clean-UP System Kit (Promega Corporation, Madison, WI, USA), according to the manufacturer’s instructions. The samples were sequenced using the BigDye Terminator v3.1 Cycle Sequencing Kit (Thermo Scientific, Waltham, MA, USA).

### 2.5. Multiplex PCR for Detection of the Three Infections

As a positive control for multiplex PCR (M-PCR) for the simultaneous detection of *C. trachomatis*, *T. vaginalis*, and *N. gonorrhoeae*, a gene block was constructed from the sequences corresponding to the target regions of each primer pair using the Primer-BLAST (https://www.ncbi.nlm.nih.gov/tools/primer-blast/ (accessed on 1 September 2025)) (sequence IDs: NC_002946.2; NC_000117.1; NW_001820777.1) (Table 2). Spacers with 10 nucleotides were added between each region. Serial dilutions of the gene block were performed, with an initial concentration of 10 ng/μL and a final concentration of 0.07 ng/μL. Nuclease-free H_2_O was used for dilution.

The multiplex PCR (M-PCR) protocol was based on Gimenes et al. [44], with modifications. For each sample, 0.8 μL of MgCl_2_, 4.2 μL of H_2_O, 10 μL of PCR Master Mix (Promega Corporation, Madison, WI, USA), 0.2 μL of each primer, and 4 μL of DNA were added. The parameters used in PCR were: initial denaturation at 94 °C for 5 min; 35 cycles at 94 °C for 45 s, annealing at 62 °C for 30 s, extension at 72 °C for 30 s, and 72 °C for 5 min for final extension. The M-PCR product was subjected to electrophoresis in 1.5% agarose gel.

### 2.6. Bioinformatics and Statistical Analysis

The data obtained after sequencing were processed using the Pregap4 and Gap4 programs (Staden Package) [46] to assess the quality and assembly of the contigs. Subsequently, the sequences were aligned with those in the GenBank database (NCBI) using BLASTN [47] (https://blast.ncbi.nlm.nih.gov/ (accessed on 22 May 2020)) to confirm the microorganisms. Statistical analyses were performed using SPSS^®^ software (Statistical Package for the Social Sciences) version 30.0 (IBM Corp., Armonk, NY, USA). Fisher’s exact test was used to assess possible associations between categorical variables, with *p*-values < 0.05 considered statistically significant.

## 3. Results

The study population comprised 155 HPV-positive women living with HIV, and were subsequently divided into two groups: (1) women who were HIV-HPV positive only, and (2) women who were HIV-HPV positive with an additional sexually transmitted infection (STI).

The majority were between 25 and 35 years of age (34.2%), single (56.8%), of self-reported mixed/brown ethnicity (60.2%), had incomplete elementary education (44.2%), and reported a monthly income equivalent to one to two minimum wages (62%). Most participants reported having a steady partner (69.6%), of whom 49.5% were HIV positive, never using illicit drugs (84.8%), and consistent condom use (48.3%). Additionally, 69.3% reported HIV acquisition from a previous partner. Clinically, most women reported being on antiretroviral therapy (ART) (95.4%), had normal CD4+ T-cell counts (49.3%), and maintained an undetectable viral load (66.7%) (Supplementary Table S1).

In the univariate analysis, variables such as age group, marital status, drug use, and condom use showed no statistically significant differences between HIV-HPV positive and HIV-HPV-STI positive groups (*p* > 0.05). Conversely, the partner’s HIV serological status was significantly associated with the group distribution (*p* = 0.026) (Supplementary Table S1).

Samples were tested for the presence of *Chlamydia trachomatis*, *Trichomonas vaginalis*, and *Neisseria gonorrhoeae* DNA. During the standardization of the conventional PCR assay for *C. trachomatis* detection, annealing temperature gradients of 52 °C, 54 °C, 55 °C, and 56 °C were tested. The best amplification was obtained at 55 °C, with no nonspecific bands observed. After optimization, all 155 samples were analyzed, of which 12 (7.74%) tested positive for *C. trachomatis* and 10 (6.45%) for *T. vaginalis*, later confirmed by sequencing. None of the samples tested positive for *N. gonorrhoeae.*

**Figure 1:**
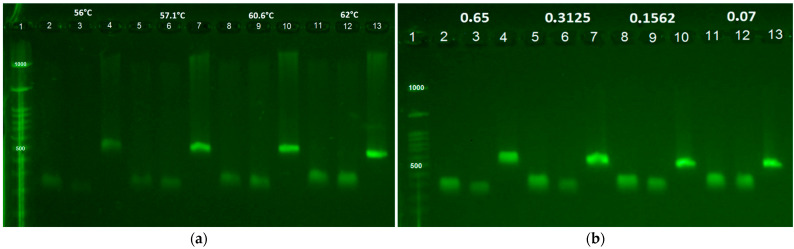
(**a**) Agarose gel electrophoresis (1.5%) of M-PCR products. Lane 1: molecular weight marker; Lanes 2–4: positive samples for *Trichomonas vaginalis* (70 bp), *Neisseria gonorrhoeae* (162 bp), and *Chlamydia trachomatis* (361 bp), respectively, using an annealing temperature of 56 °C; Lanes 5–7: positive samples for *T. vaginalis* (170 bp), *N. gonorrhoeae* (162 bp), and *C. trachomatis* (361 bp), respectively, using an annealing temperature of 57.1 °C; Lanes 8–10: positive samples for *T. vaginalis* (170 bp), *N. gonorrhoeae* (162 bp), and *C. trachomatis* (361 bp), respectively, using an annealing temperature of 60.6 °C; Lanes 11–13: positive samples for *T. vaginalis* (170 bp), *N. gonorrhoeae* (162 bp), and *C. trachomatis* (361 bp), respectively, using an annealing temperature of 62 °C. (**b**) Agarose gel electrophoresis (1.5%) of M-PCR products with the gene block. Lane 1: molecular weight marker; Lanes 2–4: positive samples for *Trichomonas vaginalis* (170 bp), *Neisseria gonorrhoeae* (162 bp), and *Chlamydia trachomatis* (361 bp), respectively, using a DNA concentration of 0.65 ng/μL; Lanes 5–7: positive samples for *T. vaginalis* (170 bp), *N. gonorrhoeae* (162 bp), and *C. trachomatis* (361 bp), respectively, using a DNA concentration of 0.31 ng/μL; Lanes 8–10: positive samples for *T. vaginalis* (170 bp), *N. gonorrhoeae* (162 bp), and *C. trachomatis* (361 bp), respectively, using a DNA concentration of 0.15 ng/μL; Lanes 11–13: positive samples for *T. vaginalis* (170 bp), *N. gonorrhoeae* (162 bp), and *C. trachomatis* (361 bp), respectively, using a DNA concentration of 0.07 ng/μL. Positive and negative controls were included during assay optimization; no clinical samples were used in these gels.

For M-PCR standardization, annealing temperatures of 56 °C, 57.1 °C, 60.6 °C, and 62 °C were evaluated, with 62 °C providing optimal amplification of the three gene block regions (Figure 1a). To establish the limit of detection (LOD) for each pathogen, DNA concentrations of 0.65 ng/μL, 0.31 ng/μL, 0.15 ng/μL, and 0.07 ng/μL were tested, with detection possible down to the lowest concentration (Figure 1b). The M-PCR method showed 100% sensitivity and 100% specificity in detecting *C. trachomatis* and *T. vaginalis* (Table 3). The multiplex assay detected all samples identified by conventional PCR, as well as two additional *C. trachomatis*-positive cases not previously identified. Combining both conventional and multiplex assays, the overall prevalence was 9.03% (14/155) for *C. trachomatis* infection and 6.45% (10/155) for *T. vaginalis*. Furthermore, one sample was positive for both *C. trachomatis* and *T. vaginalis*, corresponding to a prevalence of 0.64% (1/155).

Among the HPV-positive samples, high-risk genotypes accounted for 70.9% (108/152) of cases, with HPV-16 being the most prevalent (35.5%). The second most frequent types were HPV-31 and HPV-33, each detected in 5.9% (9/152) of cases. Additionally, the low-risk genotype HPV-6 was also identified in 5.9% of the samples. In the group of women with *C. trachomatis* or *T. vaginalis*, a high frequency of high-risk HPV types was also observed, accounting for approximately 80% of cases (16/20) (Supplementary Table S2). Notably, despite the small sample size, this group, as well as the HIV-HPV positive group, encompassed 11 of the 12 carcinogenic genotypes classified by the International Agency for Research on Cancer (IARC) [48].

Regarding cytological outcomes, among 121 results available, 81.8% (99/121) were negative for intraepithelial lesions or malignancy (NILM), while 18.2% (22/121) showed cervical abnormalities, classified according to the Bethesda system. Of these, 0.8% (1/121) was classified as ASC-US, 1.7% (2/121) as ASC-H, 5.0% (6/121) as LSIL, and 10.7% (13/121) as high-grade squamous intraepithelial lesions (HSIL). Considering the 23 samples with co-detection of *C. trachomatis* or *T. vaginalis*, cytological results were available for 15. Among these, 86.7% (13/15) presented with normal cytology (NILM) and 13.2% (2/15) were associated with high-grade lesions (HSIL). The single sample positive for both pathogens was also classified as NILM (Table 4). Fisher’s exact test indicated that no statistically significant differences were observed between the variables evaluated.

## 4. Discussion

HPV and HIV co-infection facilitates cervical carcinogenesis through complex viral interactions [49] and the influence of other cofactors, such as other STIs, aggravates the scenario [13]. The aim of this study was to establish a molecular detection strategy for *Chlamydia trachomatis*, *Trichomonas vaginalis*, and *Neisseria gonorrhoeae* and to evaluate the association of these infections with the development of cervical lesions in HPV-positive women living with HIV in Northeastern Brazil. It was possible to adapt and validate the molecular detection strategy proposed by Gimenes et al. [44] for the detection of infections of great clinical relevance in samples from women with HPV/HIV in this population, as well as to relate these results to cervical lesions. A prevalence of 9.03% was observed for *C. trachomatis* infection and 6.45% for *T. vaginalis*. A prevalence of 0.64% was observed for infection by both STIs, *C. trachomatis* and *T. vaginalis*. Among the HPV-positive women living with HIV, high-risk genotypes accounted for 70.9% of cases, with HPV-16 being the most prevalent (35.5%). Regarding cytological results, 18.2% of women had cervical lesions, and among those with co-detection of *C. trachomatis* and *T. vaginalis*, 13.2% were associated with high-grade lesions (HSIL). Our epidemiological findings represent the scenario of women with HPV-HIV coinfection and other STIs in Northeastern Brazil, and reinforce the importance of molecular screening for STIs, which can inform and guide cervical cancer prevention policies for women living with HIV.

The optimal annealing temperature for the M-PCR method was established at 62 °C, enabling the efficient generation of all three amplicons. The assay demonstrated 100% sensitivity and 100% specificity for detecting *C. trachomatis* and *T. vaginalis*, and no positive cases of *N. gonorrhoeae* were identified by either the conventional or multiplex PCR methods. The multiplex assay also exhibited a detection limit down to the lowest concentration tested and proved to be highly accurate, as it did not misidentify any pathogens. Additionally, the M-PCR method detected one sample that was positive for both *C. trachomatis* and *T. vaginalis*. Several studies have reported multiplex detection strategies for the same microorganisms [29,30,31,50]. Similarly to our findings, Rostami et al. [31] developed a multiplex PCR assay targeting *C. trachomatis*, *N. gonorrhoeae*, and *T. vaginalis* using endocervical samples, and it demonstrated 100% sensitivity and specificity for detecting *C. trachomatis* and *T. vaginalis*. However, their assay showed limitations in accurately detecting *N. gonorrhoeae*. While Rostami et al. [31] reported coinfection rates between two pathogens ranging from 0.7% to 1.7%, our study observed a slightly lower rate of 0.64%, likely influenced by the smaller sample size. Tayoun et al. [29] also detected these pathogens using real-time PCR and, consistent with our results, demonstrated a detection limit extending to the lowest concentration tested. Therefore, our multiplex assay demonstrates high sensitivity, specificity, and accuracy and is capable of detecting even low concentrations of pathogen DNA. It also proved effective in identifying coinfections, making it a viable tool for detecting STIs in women living with HIV. Thus, this technique can be broadly applied for the early diagnosis of these infections in this vulnerable population, contributing to the prevention of complications and more severe outcomes.

Several studies have reported an association between host epidemiological characteristics, such as age, number of sexual partners, and oral contraceptive use, with an increased risk of HPV persistence [51,52]. However, these findings have not been consistently replicated, and there is still no consensus regarding the risk factors that may contribute to viral persistence. In this analysis, a significant association was observed only with the partner’s HIV serological status. This finding is consistent with evidence from Guthrie et al. [53], who reported that the presence of an STI in one partner strongly predicts infection in the other, underscoring the influence of partner-related factors on STI transmission dynamics. Similarly, Mbulawa et al. [54] demonstrated that HIV coinfection in one or both sexually active partners increases HPV prevalence, further supporting the relevance of partner characteristics in infection risk. In addition, the authors observed that HIV-positive or serodiscordant couples exhibited a greater sharing of multiple HPV genotypes, reinforcing the impact of HIV on HPV transmission patterns. Most of participants reported being on antiretroviral therapy (ART), with normal CD4 counts and undetectable viral load. Previous studies have demonstrated that low CD4 counts (<200 cells/μL) [55,56,57], detectable viral load (>50 copies/mL) [58,59], and shorter duration of ART use [60,61] are important risk factors for HPV infection and progression to precancerous lesions. Nevertheless, individuals living with HIV, even when receiving effective ART and maintaining high CD4 counts, remain at increased risk for HPV infection, and have higher prevalence of HR-HPV infections, suggesting that additional factors beyond immune suppression contribute to this vulnerability [62].

A systematic review by Tadese et al. [5] showed that the prevalence of HPV in women living with HIV varies globally from 20% to 98%. A study by Nyasenu et al. [63] identified a prevalence of 22.2% of HPV in women living with HIV. Similarly, our study found that among women living with HIV, HPV infection had a prevalence of 57.4%. In relation to women living with HIV-HPV infection and other STIs, this study identified *C. trachomatis* and *T. vaginalis* infections with a prevalence of 9.03% and 6.45%, respectively. Similarly, a study by Rodrigues et al. [13] reported a prevalence of 19.5% for *C. trachomatis* and 9.8% for *T. vaginalis* in HPV-HIV coinfected women. In addition, a prevalence of 10% was identified for co-infection with both STIs, while our study reported only 0.64%. Regarding *N. gonorrhoeae*, no positive cases were detected in our cohort. Rodrigues et al. [13] reported a prevalence of 4.9% in their study population, and Mukanyangezi et al. [64] showed that HIV-positive women had a higher proportion of gonorrhea compared to HIV-negative women. The absence of *N. gonorrhoeae* in our study may therefore reflect a lower circulation of this pathogen in the region or differences in population characteristics and screening dynamics. These findings emphasize the importance of detection strategies in women with HPV-HIV coinfection, as regional variations in STI prevalence highlight the need for more specific and targeted screening and prevention measures.

Regarding HPV types, our study identified a prevalence of 60.5% of HR-HPV in women living with HIV. Similarly, Blossom et al. [65] reported HR-HPVs in all cases in this population, with type 16 present in approximately 20% of cases, while Thorsteinsson et al. [66] identified HR-HPVs in 28% of cases, with type 16 present in approximately 5% of them. Musa et al. [67] reported a prevalence of 44.9%, while Teixeira et al. [68] identified 31.1%, with type 16 ranking as the third most frequent (19.4%). Cambrea et al. [69] reported a frequency of 47.5% of HR-HPV in this population, with type 16 present in only 5% of samples. In contrast, our study found type 16 to be the most frequent, detected in 30.3% of cases in women living with HIV. In the context of HIV-HPV infection and other STIs coinfections, Rodrigues et al. [13] identified multiple infections in all analyzed samples, with HR-HPVs present in 31.4%. In this study, however, HR-HPVs were detected in only 10.5% of HIV-HPV infection and other STI cases. These findings reinforce that HR-HPV infection in women living with HIV increases the risk of cervical lesion progression [7], a risk further intensified by the presence of other STIs, such as *C. trachomatis* and *T. vaginalis*.

In this study, 81.1% of HIV-HPV coinfected women showed no cervical abnormalities. Similarly, Jary et al. [70] also reported a lower occurrence of cervical lesions among HIV-positive women, with abnormalities more frequently observed in HIV-negative women. This result, however, contrasts with the literature, which generally reports a higher frequency of precancerous cervical lesions among women living with HIV, with a 1.5- to 3-fold increased risk compared to HIV-negative women [5]. Among the 18.9% of women who presented abnormal cytology in our study, 5.7% were classified as LSIL and 10.4% as HSIL. These findings differ from those reported by Freitas et al. [71], who observed prevalence of 18.8% for LSIL and 6.2% for HSIL. Even more pronounced differences were described by Monteiro et al. [72], whose results showed rates of 39.4% for LSIL and 45.4% for HSIL.

Among women living with HIV-HPV coinfection and other STIs, 86.7% presented normal cytology (NILM), while 13.2% exhibited HSIL. Although our study did not find statistically significant differences between the variables, evidence suggests that some co-infections may influence HPV persistence and cervical lesion progression in this population. Raffone et al. [73] demonstrated that *Trichomonas vaginalis* infection acts as a risk factor for the progression of CIN1/LSIL specifically in HPV-positive women living with HIV, indicating that *T. vaginalis* may contribute to lesion worsening in immunocompromised hosts. In contrast, Mukanyangezi et al. [64] did not evaluate lesion progression in HIV-positive women with *T. vaginalis* but identified that a history of *N. gonorrhea* was positively associated with HR-HPV infection in this group. These findings reinforce the potential relevance of *T. vaginalis* and *N. gonorrhoeae* as co-factors capable of modifying the natural history of HPV infection.

Despite these observations, studies that simultaneously evaluate HPV, HIV, and other STIs are still scarce in Brazil. Although previous investigations have evaluated HPV and *C. trachomatis* in women living with HIV, none have reported triple infections or explored their potential synergistic impact on the development of cervical lesions. The scarcity of studies addressing these simultaneous infections, particularly those incorporating cytology or histology, represents a significant gap in understanding how multiple pathogens interact to influence cervical carcinogenesis in immunocompromised women. Considering that HIV, HPV, *C. trachomatis*, *T. vaginalis*, and *N. gonorrhoeae* each independently promote inflammation, epithelial disruption, and viral persistence, investigating their combined effects is biologically plausible and urgently needed [74,75,76].

Although the relevance of the findings reported in this study, it has some limitations that should be acknowledged. First, the lack of evaluation of clinical symptoms, which reduces our ability to assess the clinical relevance of detected infections. The study also had limited statistical power to detect associations with cervical lesions, and some behavioral information relied on self-reported data, which is subject to recall and reporting bias. The evaluation of *N. gonorrhoeae* was restricted due to the absence of positive cases, preventing further analysis. Finally, the study design may introduce selection bias, as data were obtained from a single center and from a specific population of women living with HIV, which may limit the generalizability of the findings.

Therefore, given the current gaps in understanding the dynamics of STI, HPV, and HIV coinfections, further multicenter studies are needed to assess the prevalence of these coinfections in diverse populations of HPV-positive women living with HIV, comparing regional patterns, clinical outcomes, and differences in vulnerability. Such investigations would help clarify the broader impact of STI coinfections in HPV-positive women living with HIV and inform targeted strategies for prevention and early clinical management.

## 5. Conclusions

Therefore, the high prevalence of high-risk HPV types observed in this cohort highlights the increased susceptibility of women living with HIV to high-risk HPV types. The results reinforce the importance of molecular diagnostics and routine screening for genital infections, given the potential for coinfections with multiple pathogens. Overall, this study highlights the need to strengthen preventive strategies and expand access to health services for women living with HIV, with the aim of reducing the risk of adverse outcomes such as cervical cancer. These findings highlight the importance of implementing new health measures based on improved guidelines for STI screening in HIV-positive women, integration of molecular diagnostic methods into routine care, improved follow-up systems for monitoring cervical lesions, and targeted health education programs to support early detection and treatment of lesions, in order to reduce the risk of adverse outcomes, such as cervical cancer.

## Figures and Tables

**Table 1:** Sequences of primers used for the detection of *C. trachomatis* by conventional PCR.

Primer	Sequence (5′-3′)	Fragment Size (bp)	Reference
CTP1	TAGTAACTGCCACTTCATCA	201	
CTP2	TTCCCCTTGTAATTCGTTGC		[43]
PL61	AGAGTACATCGGTCAACG	130	
PL62	TCACAGCGGTTGCTCGAAGCA		

**Table 2:** Sequence of primers used for detection of *N. gonorrhoeae*, *T. vaginalis*, and *C. trachomatis* by M-PCR.

Primer	Sequence (5′-3′)	Fragment Size (bp)	Reference
*N. gonorrhoeae F*	CGGCAGCATTCAATTTGTT	162	
*N. gonorrhoeae R*	AAAAAGCCGCCATTTTTGTA		
*T. vaginalis F*	CCAGAAGTGGGCTACACACC	170	[45]
*T. vaginalis R*	ATACCAAGGCCGGAAGCAC		
*C. trachomatis F*	TCTTTTTAAACCTCCGGAACCCACTT	361	
*C. trachomatis R*	GGATGGCATCGCATAGCATTCTTTG		

**Table 3:** Sensitivity and specificity of different methods used for detection of *C. trachomatis* and *T. vaginalis*.

Organism	Method	Sensitivity % (No. of True Positive/ No. of Infected Women)	Specificity % (No. of True Negative/ No. of Non-Infected Women)
*C. trachomatis*	Conventional PCR	85.71% (12/14)	98.60% (141/143)
	M-PCR	100% (14/14)	100% (141/141)
*T. vaginalis*	Conventional PCR	100% (10/10)	100% (145/145)
	M-PCR	100% (10/10)	100% (145/145)

M-PCR: multiplex PCR.

**Table 4:** Cytological diagnoses among HIV-HPV positive women and HIV-HPV + other STI (*C. trachomatis* and/or *T. vaginalis*) positive women (*n* = 121).

Cytology	N (%)	HIV-HPV (%)	HIV-HPV + Other STI (%)	*p*-Value
NILM	99 (81.8)	86 (81.1)	13 (86.7)	
ASC-US	1 (0.8)	1 (0.9)	0 (0.0)	
ASC-H	2 (1.7)	2 (1.9)	0 (0.0)	0.909
LSIL	6 (5.0)	6 (5.7)	0 (0.0)	
HSIL	13 (10.7)	11 (10.4)	2 (13.2)	

NILM: Negative for intraepithelial lesions or malignancy, ASC-US: atypical squamous cells of undetermined significance, ASC-H: atypical squamous cells in which a high-grade squamous intraepithelial lesion cannot be excluded, LSIL: low-grade squamous intraepithelial lesions, HSIL: high-grade squamous intraepithelial lesions.

## Data Availability

The raw data supporting the conclusions of this article will be made available by the authors on request.

## Supplementary Materials

The following supporting information can be downloaded at: https://www.mdpi.com/article/10.3390/tropicalmed10120354/s1, Table S1: Association between epidemiological characteristics among HIV-HPV positive women and HIV-HPV + other STI (*C. trachomatis* and/or *T. vaginalis*) positive women; Table S2: Distribution of HPV types among HIV-HPV positive women and HIV-HPV + other STI (*C. trachomatis* and/or *T. vaginalis*) positive women.
