# Supplementary material for: Multi-Target Molecular Detection of Sexually Transmitted Infections in Women Living with HIV in Northeastern Brazil
Source: Trop Med Infect Dis. 2025 Dec 18;10(12):354. doi: 10.3390/tropicalmed10120354 (PMC12737351; doi:10.3390/tropicalmed10120354)
Supplement: Supplementary file 1 [file tropicalmed-10-00354-s001.zip › tropicalmed-3934252-supplementary.pdf]

SUPPLEMENTARY MATERIALS:

Supplementary Table S1 and S2

Table S1: Association between epidemiological characteristics among HIV-HPV positive women and HIV-HPV + other STI (*C. trachomatis* and/or *T. vaginalis*) positive women.

	N (%)	HIV-HPV (%)	HIV-HPV + other STI (%)	p-Value
**Age (years)**	152 (100%)	132 (100%)	20 (100%)	
<25	24 (15.8)	21 (15.9)	3 (15.0)	
25-35	52 (34.2)	47 (35.6)	5 (25.0)	
36-45	48 (31.6)	40 (30.3)	8 (40.0)	0.759
46-55	23 (15.1)	19 (14.4)	4 (20.0)	
>55	5 (3.3)	5 (3.8)	0 (0.0)	
**Marital status**	118 (100%)	102 (100%)	16 (100%)	
Single	67 (56.8)	56 (54.9)	11 (68.8)	
Married	33 (28.0)	30 (29.4)	3 (18.8)	0.853
Divorced	8 (6.8)	7 (6.9)	1 (6.2)	
Widow	10 (8.4)	9 (8.8)	1 (6.2)	
**Ethnicity**	113 (100%)	97 (100%)	16 (100%)	
White	12 (10.6)	9 (9.3)	3 (18.8)	
Black	32 (28.3)	28 (28.9)	4 (25.0)	0.583
Brown	68 (60.2)	59 (60.8)	9 (56.2)	
Indigenous	1 (0.9)	1 (1.0)	0 (0.0)	
**Education**	138 (100%)	121 (100%)	17 (100%)	
Illiterate	4 (2.9)	2 (1.7)	2 (11.8)	
Incomplete Elementary Education	61 (44.2)	53 (43.8)	8 (47.1)	
Complete Elementary Education	16 (11.6)	15 (12.4)	1 (5.9)	
Incomplete High School	16 (11.6)	13 (10.7)	3 (17.6)	0.321
Complete High School	35 (25.4)	32 (26.5)	3 (17.6)	
Incomplete Higher Education	1 (0.7)	1 (0.8)	0 (0.0)	
Complete Higher Education	5 (3.6)	5 (4.1)	0 (0.0)	
**Economic Situation**	137 (100%)	120 (100%)	17 (100%)	
No income	9 (6.6)	9 (7.5)	0 (0.0)	
Less than 1 salary	26 (19.0)	22 (18.3)	4 (23.5)	0.614
From 1 to 2 salaries	85 (62.0)	75 (62.5)	10 (58.8)	
From 2 to 5 salaries	17 (12.4)	14 (11.7)	3 (17.7)	
**Marital Bond**	138 (100%)	121 (100%)	17 (100%)	
Regular partner	96 (69.5)	85 (70.3)	11 (64.7)	
Occasional partner	7 (5.1)	5 (4.1)	2 (11.8)	0.537
Fixed and occasional partner	3 (2.2)	3 (2.5)	0 (0.0)	
Does not have	32 (23.2)	28 (23.1)	4 (23.5)	
**Partner's HIV serological status**	91 (100%)	78 (100%)	13 (100%)	
Regular HIV+ partner	45 (49.5)	43 (55.1)	2 (15.4)	
Occasional HIV+ partner	2 (2.2)	1 (1.3)	1 (7.7)	
Non-carrier fixed partner	39 (42.8)	30 (38.5)	9 (69.2)	0.026
Non-carrier occasional partner	4 (4.4)	3 (3.8)	1 (7.7)	
Neither of them is a carrier	1 (1.1)	1 (1.3)	0 (0.0)	

**Drug Use**	138 (100%)	121 (100%)	17 (100%)	
Non-intravenous drug user	7 (5.1)	7 (5.8)	0 (0.0)	
Former drug user	5 (3.6)	5 (4.1)	0 (0.0)	0.566
Have tried drugs	9 (6.5)	7 (5.8)	2 (11.8)	
Never used drugs	117 (84.8)	102 (84.3)	15 (88.2)	
**Condom Use**	118 (100%)	101 (100%)	17 (100%)	
Always	57 (48.3)	49 (48.5)	8 (47.2)	
Most of the time	11 (9.3)	8 (7.9)	3 (17.6)	
Sometimes	22 (18.6)	19 (18.8)	3 (17.6)	0.77
Rarely	3 (2.6)	3 (3.0)	0 (0.0)	
Never	25 (21.2)	22 (21.8)	3 (17.6)	
**HIV Exposure**	114 (100%)	102 (100%)	12 (100%)	
Current HIV+ partner	33 (28.9)	32 (31.4)	1 (8.3)	
Previous HIV+ partner	79 (69.3)	69 (67.6)	10 (83.4)	0.056
Vertical transmission	2 (1.8)	1 (1.0)	1 (8.3)	
**ART Use**	152 (100%)	132 (100%)	20 (100%)	
Yes	145 (95.4)	125 (94.7)	20 (100)	0.365
No	7 (4.6)	7 (5.3)	0 (0.0)	
**ART Time of Use (months)**	143 (100%)	125 (100%)	18 (100%)	
1-6	36 (25.2)	34 (27.2)	2 (11.0)	
7-24	26 (18.2)	21 (16.8)	5 (27.8)	
25-60	33 (23.0)	30 (24.0)	3 (16.7)	0.321
61-120	24 (16.8)	19 (15.2)	5 (27.8)	
>120	24 (16.8)	21 (16.8)	3 (16.7)	
**CD4 Count**	144 (100%)	126 (100%)	18 (100%)	
Very low (< 200)	14 (9.7)	12 (9.5)	2 (11.1)	
Low (200 - < 500)	58 (40.3)	53 (42.1)	5 (27.8)	0.559
Normal (500 - 1500)	71 (49.3)	60 (47.6)	11 (61.1)	
High (> 1500)	1 (0.7)	1 (0.8)	0 (0.0)	
**Viral Load**	147 (100%)	128 (100%)	19 (100%)	
Undetectable	98 (66.6)	82 (64.1)	16 (84.2)	
50 - <1.000	17 (11.6)	17 (13.3)	0 (0.0)	
1.000 - < 10.000	11 (7.5)	11 (8.6)	0 (0.0)	0.242
10.000 - < 100.000	12 (8.2)	10 (7.8)	2 (10.5)	
> 100.000	9 (6.1)	8 (6.2)	1 (5.3)	

HIV: human immunodeficiency virus, HPV: human papillomavirus, STI: sexually transmitted infection, ART: antiretroviral therapy, CD4: T cell count

Table S2: Distribution of HPV types among HIV-HPV positive women and HIV-HPV + other STI (*C. trachomatis* and/or *T. vaginalis*) positive women.

HPV genotypes	N	%	HPV-HIV	%	HPV-HIV + other STI	%
**Low-risk types**	30	19.8	27	17.7	3	2.1
HPV-6	9	5.9	9	5.9	0	0.0
HPV-11	4	2.6	3	1.9	1	0.7
HPV-44	1	0.7	1	0.7	0	0.0
HPV-54	1	0.7	1	0.7	0	0.0
HPV-61	1	0.7	0	0.0	1	0.7
HPV-62	4	2.6	4	2.6	0	0.0
HPV-81	4	2.6	4	2.6	0	0.0
HPV-86	1	0.7	1	0.7	0	0.0
HPV-87	1	0.7	1	0.7	0	0.0
HPV-114	4	2.6	3	1.9	1	0.7
**Possibly high-risk types**	14	9.3	13	8.6	1	0.7
HPV-30	2	1.3	2	1.3	0	0.0
HPV-53	6	3.9	6	4.0	0	0.0
HPV-66	5	3.3	4	2.6	1	0.7
HPV-67	1	0.7	1	0.7	0	0.0
**High-risk types**	108	70.9	92	60.3	16	10.6
HPV-16	54	35.5	46	30.3	8	5.2
HPV-18	4	2.6	4	2.6	0	0.0
HPV-31	9	5.9	8	5.2	1	0.7
HPV-33	9	5.9	8	5.2	1	0.7
HPV-35	8	5.3	7	4.6	1	0.7
HPV-45	8	5.3	8	5.2	0	0.0
HPV-51	1	0.7	1	0.7	0	0.0
HPV-52	3	1.9	2	1.3	1	0.7
HPV-56	4	2.6	3	1.9	1	0.7
HPV-58	7	4.6	4	2.6	3	1.9
HPV-59	1	0.7	1	0.7	0	0.0

HIV: human immunodeficiency virus, HPV: human papillomavirus, STI: sexually transmitted infection.
